# Thyrotoxicosis and Pregnancy

**DOI:** 10.1371/journal.pmed.0020370

**Published:** 2005-12-27

**Authors:** Petros Perros

## Abstract

A 35-year-old woman presented with a neck swelling after a missed abortion. Her thyroid function tests were in the thyrotoxic range. Perros discusses the further investigation and management of this patient.

## DESCRIPTION of CASE

A 35-year-old woman became aware of a swelling in her neck about ten weeks before referral. She had been trying to conceive for ten months. She had had a missed abortion five months earlier. Because of the neck swelling, her family doctor arranged thyroid function tests, which were in the thyrotoxic range on two occasions five weeks apart: serum free thyroxine, 26 and 28 pmol/l (normal range, 11–23); serum free tri-iodothyronine, 10.9 and 11 pmol/l (normal range, 3.5–6.5); serum thyroid-stimulating hormone (TSH), <0.05 mU/l (normal range, 0.3–4.1).

Her previous medical history included a partial thyroidectomy for thyrotoxicosis at the age of 24. Other than a goitre, she had no symptoms except increased appetite and a slight tremor, which she had been aware of for about eight weeks. Following the missed abortion, she had two normal menstrual periods. Her only medication was folic acid supplements. She worked part-time and had a two-and-a-half-year-old child.

**Figure 1:**
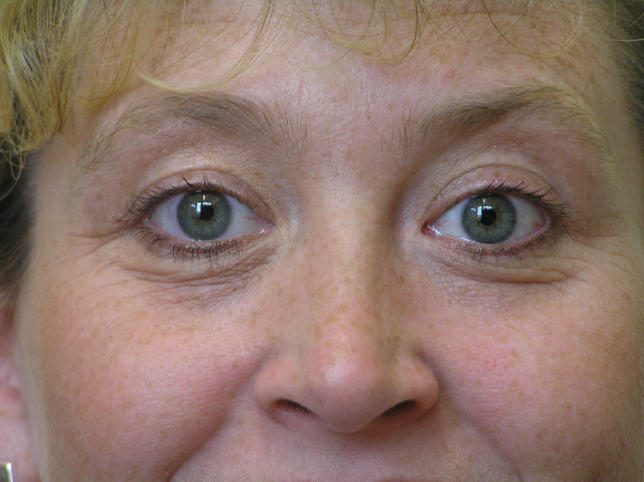
The Patient's Appearance at the Time of Presentation with Recurrent Thyrotoxicosis Note that there are no signs of TAO, but the patient has minimal upper lid retraction (the upper lid should normally be halfway between the limbus and the pupil).

On examination she was of average weight. Her hands were warm and moist. There was a fine tremor. A previous thyroidectomy scar was noted. The right lobe of the thyroid was palpable and felt smooth. There was a bruit over the right thyroid lobe on auscultation. She had lid retraction and lid lag but no other signs suggestive of thyroid-associated ophthalmopathy (TAO) (Figure 1). Her pulse rate was 100 beats per minute and regular. Her blood pressure was 150/70 mm Hg. The rest of the examination was normal.

### What Is the Cause of Her Thyrotoxicosis?

Thyrotoxicosis is no more than a descriptor for a pattern of biochemical abnormalities. Before considering treatment, it is the clinician's task to define the underlying cause, as an accurate diagnosis is an essential guide to the most appropriate treatment (Box 1).

**Box 1. Causes of Thyrotoxicosis**

**Common causes of thyrotoxicosis in a young female**

- Graves disease
- Thyroiditis
- Toxic multinodular goitre
- Toxic adenoma
- Iodine excess

**Other rare causes of thyrotoxicosis**

- Hyperemesis gravidarum
- Choriocarcinoma
- TSH-producing pituitary adenoma
- Iatrogenic thyrotoxicosis
- Factitious thyrotoxicosis
- Struma ovarii
- Metastatic follicular thyroid cancer
- Thyroid hormone resistance syndrome

The most likely causes in this case were Graves disease, thyroiditis, toxic multinodular goitre (TMNG), and toxic adenoma. The hallmark of TMNG or toxic adenoma is the presence of one or more palpable thyroid nodules. In this case the patient had previously undergone a partial thyroidectomy and a vascular thyroid remnant was palpable on the right thyroid lobe. Post-partum thyroiditis occurs within 12 months of childbirth; a variant of this condition occurs after miscarriage. In this patient's case post-partum thyroiditis was unlikely because her previous pregnancy was 2.5 years earlier; however, the miscarriage five months earlier may have been relevant. Viral thyroiditis is usually preceded by an upper respiratory tract infection and the thyroid gland is tender to touch; the absence of these features makes viral thyroiditis unlikely. “Silent” thyroiditis may present in this way and was a possibility here.

**Figure 2:**
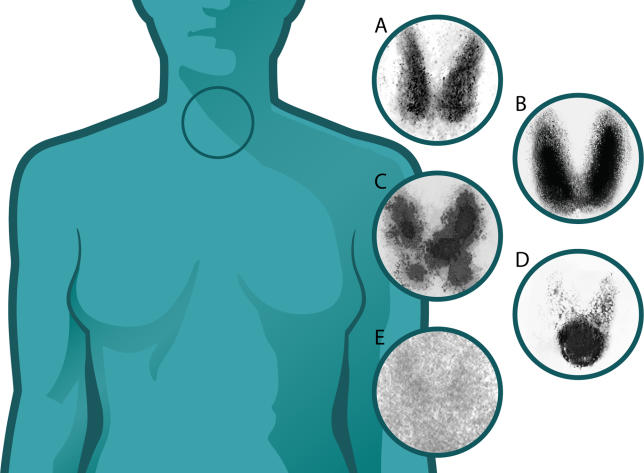
Technetium 99 Thyroid Uptake Scans (A) Normal. (B) Graves disease: diffuse increased uptake in both thyroid lobes. (C) TMNG: “hot” and “cold” areas of uneven uptake. (D) Toxic adenoma: increased uptake in a single nodule with suppression of the surrounding thyroid. (E) Thyroiditis: decreased or absent uptake. (Image: Giovanni Maki)

Laboratory tests that may help differentiate between the different causes of thyrotoxicosis include a radiolabelled technetium or iodide thyroid scan (Figure 2), and measurement of anti–thyroid peroxidase (TPO) antibodies, TSH receptor antibodies, and inflammatory markers (Table 1). The thyrotoxic phase of thyroiditis is usually followed by spontaneous euthyroidism and in some cases hypothyroidism. Repeating thyroid function tests within a few weeks of the first set may identify cases of thyroiditis.

**Table 1:**
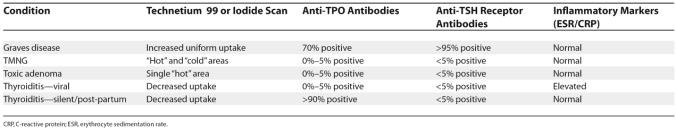
Diagnostic Tests for Identifying the Cause of Thyrotoxicosis

CRP, C-reactive protein; ESR, erythrocyte sedimentation rate.

In this case the prolonged time course of thyrotoxicosis, the presence of a vascular thyroid remnant, the persistently thyrotoxic thyroid function tests, and the elevated serum levels of TSH receptor antibodies (62 U/l; reference range, 0–10) were in favour of a diagnosis of recurrent Graves disease.

### What Are the Effects of Thyrotoxicosis on Fertility and Risk of Abortion?

Menstrual irregularities occur in about 20% of thyrotoxic women [1]. Infertility is common in women with thyrotoxicosis even when they maintain ovulatory cycles [1]. Thyrotoxicosis also increases the risk of miscarriage to 26% [2].

### How Should This Patient Be Treated?

There are three treatment options for thyrotoxicosis due to Graves disease: radioiodine (^131^I) therapy, thyroidectomy, and anti-thyroid drugs [3]. ^131^I therapy is safe and effective, but pregnancy should be deferred for 4–6 months after treatment as there are theoretical risks of fetal abnormalities. Most national regulatory authorities recommend avoidance of close contact with adults for a few days and with children and pregnant women for 2–3 weeks. ^131^I therapy was not appropriate for this patient because she wished to proceed with pregnancy as soon as possible and she had a two-and-a-half-year-old child, who would be difficult to care for after ^131^I therapy.

A second thyroidectomy is worthy of consideration, but involves general anaesthesia and a period of recuperation of a few weeks and therefore disruption of family and professional life. The risks of damage to the recurrent laryngeal nerves and parathyroid glands after a second thyroidectomy are considerably greater than after a first operation and are of the order of 5%–10%. Because of these considerations, thyroidectomy was not felt to be a suitable option.

Anti-thyroid drugs (carbimazole, methimazole, and propylthiouracil) restore euthyroidism within a few weeks of initiation of treatment [4]. Minor side effects (such as skin rashes) occur in about 5% of cases. Agranulocytosis is rare (∼0.4%), but the consequences are life threatening and all patients on anti-thyroid drugs must be made aware of this complication (Box 2). All anti-thyroid drugs have been used and are acceptable in pregnancy.

**Box 2. Patient Information Leaflet Used by the Author to Remind Patients Receiving Anti-Thyroid Drugs of the Potential Complication of Agranulocytosis**

“You have been started on a drug called Carbimazole/Methimazole/Propylthiouracil to control the activity of your thyroid gland. This is important treatment and Carbimazole/Methimazole/Propylthiouracil is a well established drug that has been used for many years. The great majority of people treated with Carbimazole/Methimazole/Propylthiouracil have no problems whatsoever.

“Some people occasionally develop a rash—if this happens please consult your doctor as soon as possible; you need not discontinue the drug unless he/she tells you to do so.

“More rarely, Carbimazole/Methimazole/Propylthiouracil affects white cells in the blood, in which case you would be likely to develop a very severe sore throat and to feel ill with a fever. If this happens while you are on Carbimazole/Methimazole/Propylthiouracil treatment you must attend either your family doctor or the hospital on the same day, to have your blood count checked. Take no more tablets until the blood count has been checked. If your white blood count is normal you can carry on with the Carbimazole/Methimazole/Propylthiouracil. If your white blood count is abnormal your family doctor or the hospital will need to deal with this problem urgently.

“Please keep this with you in case you need to show it to your doctor.”

Congenital anomalies have been reported in association with anti-thyroid drugs, but the increase in risk above background is very marginal. The risks of aplasia cutis and choanal and oesophageal atresia may be slightly lower with propylthiouracil than with other anti-thyroid drugs (choanal and oesophageal atresia, scalp defects, minor facial anomalies, and psychomotor delay compose an embryopathy implicated with methimazole use). But because the evidence is inconclusive and the additional risk minimal, all three drugs are widely used in pregnancy. The lowest dose of anti-thyroid drug that maintains euthyroidism should be used in women who wish to become or are already pregnant, in order to avoid fetal hypothyroidism and fetal goitre formation.

In this case propylthiouracil was used initially, at a dose of 50 mg four times per day. The patient was advised to take contraceptive measures until euthyroidism. Four weeks later her thyroid function tests had improved: serum free thyroxine, 13 pmol/l; serum total tri-iodothyronine, 2.5 nmol/l (normal range, 1.34–2.73); serum TSH, <0.05 mU/l. The dose of propylthiouracil was reduced to 25 mg four times per day, and the patient was advised that she could start trying to conceive.

### What Are the Risks of TAO?

TAO is a complication that many patients fear. It can be disfiguring and difficult to treat [3]. If there are no clinical features of TAO at presentation, the risk of developing it in future is approximately 15%. Smoking is an important predisposing factor. As this patient was a non-smoker the probability of developing TAO is less than 10%.

### What Monitoring Is Required during Pregnancy?

The dose of anti-thyroid drug usually needs to be decreased during pregnancy, and often Graves disease remits completely and the medication can be withdrawn. This is probably due to the overall immunosuppressive effect of pregnancy.

Monitoring of free thyroid hormone concentrations is of paramount importance during pregnancy and should be performed every 4–6 weeks, or more frequently if thyroid status is changing. The biochemical target is to achieve and maintain maternal serum free thyroxine levels at or slightly above the upper limit of normal, using the lowest dose of anti-thyroid drug possible. TSH receptor antibodies should be measured in the third trimester because positivity is predictive of neonatal thyrotoxicosis [5].

When the mother (as in this case) has a functioning thyroid gland or remnant in situ, maternal thyroid function mirrors that of the fetus. If there are concerns about fetal thyrotoxicosis (e.g., because maternal hyperthyroidism proves difficult to control), fetal heart rate monitoring should be undertaken. A persistent fetal tachycardia greater than 160 beats per minute is suggestive of fetal thyrotoxicosis. In cases where fetal thyrotoxicosis is diagnosed, monitoring of fetal growth and fetal goitre by ultrasound is imperative. In most cases the fetus can be treated satisfactorily by adjusting the dose of anti-thyroid drug in the mother and by following the fetal response clinically and by ultrasound.

### What Are the Risks to the Fetus in a Woman with Graves Disease?

Poor control of maternal hyperthyroidism is associated with significant obstetric complications including miscarriage (26%), low birth weight, prematurity, (pre-)eclampsia, and possibly congenital malformations [6]. After the fetal thyroid matures (from 20 weeks of gestation onwards), maternal TSH receptor antibodies may act on the fetal thyroid to cause fetal thyrotoxicosis and goitre. The risk of fetal thyrotoxicosis is about 1% of all pregnancies in women with Graves disease, and if untreated, fetal mortality may be as high as 24%. Overtreatment may lead to hypothyroidism in the fetus, which is associated with subtle neurocognitive deficits later on in life, particularly if the hypothyroidism occurs in the first trimester [7]. Fetal goitre can develop as a result of fetal thyrotoxicosis or fetal hypothyroidism and in severe cases can obstruct labour.

### What Are the Risks of Recurrence of Thyrotoxicosis after Delivery?

The risk of relapse of maternal thyrotoxicosis is high in the post-partum period (up to 80%), and close monitoring is required. Anti-thyroid drugs can be used safely during breastfeeding [8].

### Prenatal Counselling of Women with Graves Disease

Pregnancy is a common concern among women of childbearing age who are receiving treatment for Graves disease. Some women may elect to have definitive treatment before pregnancy, which can be either a thyroidectomy or ^131^I therapy. The advantage of these treatment options is that the risk of maternal thyrotoxicosis during pregnancy is reduced, if not eliminated. Fertility is not affected by ^131^I therapy for thyrotoxicosis, but pregnancy should be deferred for 4–6 months after ^131^I therapy, although the basis of this recommendation is largely empirical. The risk of fetal and neonatal thyrotoxicosis is not eliminated by previous thyroidectomy or ^131^I therapy. The most important advice to women who have a previous history of thyroid dysfunction is to work with their practitioner to ensure that thyroid function tests are normal at the time of conception and throughout pregnancy.

## DISCUSSION

### Aetiology of Graves Disease

Graves disease is an autoimmune condition and is mediated by stimulatory autoantibodies to the TSH receptor. There is a significant genetic component to the aetiology of Graves disease, although environmental factors and stress also seem to confer risk [3]. Typically, the thyroid gland of patients with Graves disease is diffusely enlarged and vascular.

### Toxic Multinodular Goitre

TMNG may also run in families. The pathogenesis is unknown. The disease begins with the formation of a single or few colloid nodules, and over a period of several years these become larger and more numerous. Some nodules are functioning and gradually acquire autonomy. With the passage of time serum TSH declines and may become undetectable until at a later stage serum free thyroid hormones rise. The hyperthyroidism of TMNG is usually mild and tends to occur in middle life or later. Toxic adenomas are benign neoplasms of the thyroid that are autonomous. In some cases they arise because of somatic mutations that lead to constitutive activation of the TSH receptor. As with TMNG, the hyperthyroidism tends to be mild.

### Thyroiditis

Thyroiditis is due to an inflammatory process affecting the thyroid epithelium. Unlike other causes of thyrotoxicosis, there is no increased synthesis of thyroid hormones; instead, stored thyroid hormones in colloid are released into the circulation because of the leaky epithelium. The thyrotoxic phase of thyroiditis may be followed by a hypothyroid phase a few weeks later, but as a rule the patient recovers and euthyroidism ensues without any intervention.

Thyroiditis may occur after a viral infection (referred to as subacute or De Quervain thyroiditis), in which case the patient typically has a viral sore throat, the thyroid is tender, and inflammatory markers (erythrocyte sedimentation rate and C-reactive protein) are raised. “Silent” thyroiditis is autoimmune and characterised by positive anti-TPO antibodies.

### Investigating the Cause of Thyrotoxicosis

In many cases of thyrotoxicosis the aetiology will be apparent from information that can be obtained from the history and clinical examination. In cases where there is doubt, additional investigations are indicated. Direct measurement of TSH receptor antibody levels is not widely available, but can be very valuable as modern assays are highly sensitive and specific. TSH receptor antibodies can occasionally be positive in post-partum thyroiditis (this seems to be particularly rare in Europe, though reported in North America and Japan), and in cases of doubt a thyroid scan showing no uptake of radioisotope is diagnostic of thyroiditis [9]. TSH receptor antibody measurement is indicated in pregnancy to assess the risks of fetal and neonatal thyrotoxicosis. Anti-TPO antibodies occur in a significant proportion of the normal population, and this limits the use of this test. High concentrations of anti-TPO antibodies are present in silent and post-partum thyroiditis. Radioisotope scans are useful in identifying the cause of thyrotoxicosis (Figure 2), but should be avoided in pregnancy.

### Treatment of Thyrotoxicosis

The treatment of thyrotoxicosis depends on the underlying cause. Anti-thyroid drugs are effective in Graves disease, TMNG, and toxic adenoma (Table 2), but not in thyroiditis because the latter is not associated with increased de novo synthesis of thyroid hormones.

**Table 2:**
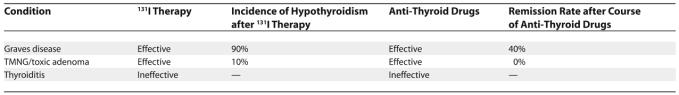
Response of Common Causes of Thyrotoxicosis to ^131^I Therapy and Anti-Thyroid Drugs

After a course of anti-thyroid drug treatment, remission may be expected in Graves disease as a result of the immunosuppressive effect of anti-thyroid drugs on synthesis of TSH receptor antibodies, but relapse is the rule in cases of TMNG or toxic adenoma.

^131^I therapy is effective for Graves disease, TMNG, and toxic adenoma. ^131^I therapy is ineffective in thyroiditis because iodine uptake is reduced or absent in this condition (Figure 2). Most patients with Graves disease develop permanent hypothyroidism after ^131^I therapy, whereas most patients with TMNG and toxic adenoma do not. ^131^I therapy is associated with a small risk of exacerbation of new development of TAO, particularly in smokers.

Thyroiditis may require symptomatic treatment with beta blockers during the thyrotoxic phase.

The type of thyroidectomy (subtotal versus total) for Graves disease as primary treatment has been the subject of controversy for some years. The argument in favour of total thyroidectomy is that the risk of recurrence of the thyrotoxicosis is eliminated, and that if performed by skilled thyroid surgeons the probability of hypoparathyroidism and vocal cord palsy is no greater than for a subtotal thyroidectomy [10]. A subtotal thyroidectomy, on the other hand, provides the best chance of any treatment for Graves disease for long-term euthyroidism without the need for thyroxine or other treatments for thyrotoxicosis.

The choice of treatment for Graves disease should be tailored to the needs of the individual patient, but also depends on local facilities, surgical expertise, and patient choice.

**Learning Points**

- Thyrotoxicosis is not a diagnosis, merely a biochemical result. An accurate clinical diagnosis encompassing the aetiology is imperative for optimal management.
- The most common cause of thyrotoxicosis in women of childbearing age is Graves disease.
- Thyrotoxicosis impairs fertility, and thyroid status should be assessed in women with secondary infertility or recurrent abortions.
- Three treatments are available for thyrotoxicosis due to Graves disease: anti-thyroid drugs, ^131^I therapy, and thyroidectomy. The right treatment is that which suits the patient's individual circumstances best.
- ^131^I therapy is an absolute contraindication in pregnancy. Anti-thyroid drugs may be used safely, and the dose should be titrated to the minimum dose that maintains normal maternal thyroid hormone levels.
- The hyperthyroidism of Graves disease usually remits after the first trimester, and anti-thyroid drugs can be withdrawn; however, relapse of maternal thyrotoxicosis in the post-partum period is common.
- Uncontrolled maternal hyperthyroidism can lead to fetal thyrotoxicosis with devastating effects on the fetus. Fetal thyrotoxicosis can be treated satisfactorily by appropriate manipulation of the maternal dose of anti-thyroid drug and careful fetal monitoring.

## Abbreviations

^131^I: radioiodine
TAO: thyroid-associated ophthalmopathy
TMNG: toxic multinodular goitre
TPO: thyroid peroxidase
TSH: thyroid-stimulating hormone
